# Chitosan Modulates Inflammatory Responses in Rats Infected with Enterotoxigenic* Escherichia coli*

**DOI:** 10.1155/2016/7432845

**Published:** 2016-12-22

**Authors:** Gang Liu, Shuai Chen, Guiping Guan, Jun Tan, Naif A. Al-Dhabi, Hongbing Wang, Veeramuthu Duraipandiyan, Jun Fang

**Affiliations:** ^1^College of Bioscience and Biotechnology, Hunan Agricultural University, Changsha, Hunan 410128, China; ^2^Institute of Subtropical Agriculture, Chinese Academy of Sciences, Scientific Observing and Experimental Station of Animal Nutrition and Feed Science in South-Central, Ministry of Agriculture, Hunan Provincial Engineering Research Center of Healthy Livestock, Key Laboratory of Agro-Ecological Processes in Subtropical Region, Changsha, Hunan 410125, China; ^3^Department of Botany and Microbiology, Addiriyah Chair for Environmental Studies, College of Science, King Saud University, P.O. Box 2455, Riyadh 11451, Saudi Arabia; ^4^Hunan Institute of Animal and Veterinary Science, Changsha, Hunan 410131, China

## Abstract

This study aims to investigate the effects of dietary chitosan (COS) on gastrointestinal pathogen resistance in mice model. For two weeks, a control group of ICR mice received a basal diet whilst the intervention group received the basal diet supplemented with 300 mg/kg COS. After two weeks, the mice fed the supplemented diet had a lower body weight. Then enterotoxigenic* Escherichia coli *(*E. coli*) was administered to the mice through oral gavage, with each mouse receiving 10^8^ CFU. At day 7 after infection, the bacterial load in the jejunum and faeces was significantly lower in the COS group than that in the control group. Moreover, the mRNA and protein levels of IL-1*β*, IL-6, IL-17, IL-18, and TNF-*α* were significantly lower in the group of mice receiving the COS diet; also the jejunal production of toll-like receptor-4 (TLR-4) was suppressed in the COS group. These results indicate the intervention influenced inflammation and controlled* E. coli* infection.

## 1. Introduction

For most people, enterotoxigenic* Escherichia coli* (ETEC) infection is merely the unpleasant and inconvenient cause of traveller's diarrhoea in many instances. ETEC is responsible for the majority of diarrhoeal disease in developing countries; according to WHO it claims approximately 380,000 lives each year [1] with children under the age of 5 years being especially vulnerable. ETEC is also prevalent amongst newborn farm animals, such as piglets [2].

ETEC infections usually emerge 1–3 days following pathogen exposure and manifest as acute watery, diarrhoea together with fever, headache, and vomiting. These symptoms typically last for 3-4 days though some people may experience diarrhoea for a week or longer [3]. ETEC colonizes the small intestine and releases enterotoxins that cause intestinal epithelial cells (IEC) to secrete fluids into the gut lumen, resulting in diarrhoea [4]. To minimise the effects of ETEC in weaning piglets, antibiotics are frequently added to their diet, with the intention of minimising infectious disease and promoting growth. However, this practice is likely to contribute to drug-resistance in pathogens by creating a reservoir of drug-resistant bacteria. These bacteria may transfer resistance genes to other pathogenic bacteria in the gut of animals and humans [5]. Also, the presence of drug residues is a concern for many consumers; therefore, the routine antibiotics in livestock diets is already banned or restricted in many countries.

Chitosan is an abundant nitrogenated polysaccharide; it is a component of fungal cell walls and exoskeletons of insects and diverse sea creatures, such as crustaceans, squid, and clams. Chitosan is derived from chitin. The immune responses of pigs [6], mice [7], rats [8], and fish [9] have been modulated by chitosan. T cells and other immune cells produce cytokines, which are essential to the immune response. In vitro studies indicate that the degree of deacetylation and molecular weight of COS are positively correlated with the extent of the anti-inflammatory effect [10]. Whilst some studies have endeavoured to evaluate the effects of chitosan on the gut bacteria of pigs and chickens [11] the effects of chitosan on inflammatory and bacterial responses during and after infection remain limited. One possible mechanism by which chitosan may operate is to disrupt* E. coli* adhesion in the jejunum.

The hypothesis that underlies this study is that dietary chitosan supplements may directly clear enterotoxigenic* E. coli *and reduce proinflammatory signals and/or increase the anti-inflammatory response during infection. We tested the hypothesis in enterotoxigenic* E. coli* infection model; the changes in the bacterial count and proinflammatory molecules were evaluated.

## 2. Mice and Diet

This experiment used 20 ICR (Institute for Cancer Research) mice aged 6 weeks; they were bred and kept at Hunan Agricultural University. Approval for all experimental procedures with the mice was granted by the Animal Care and Use Committees of Hunan Agricultural University. The mice were housed separately in pathogen-free accommodation under appropriate environmental conditions (25°C; 53% relative humidity; 12-hour light/dark cycle). They had ad lib access to an appropriate rodent diet [12] and water. Mice were randomly allocated to two groups (control *n* = 10 and chitosan intervention *n* = 10). Endotoxin free chitosan (average molecular weight < 1 kDa; degree of deacetylation > 95%) was donated by Dalian Chemical and Physical Institute (Chinese Academy of Sciences, Dalian, China). For two weeks, the control group received the basal diet and the intervention group was given a basal diet supplemented with 300 mg/kg of chitosan. The dosage and duration were determined based upon previous study [6]. Throughout the experimental period, the intake of feed and water, together with body weight gain, was monitored and recorded.

## 3. Enterotoxigenic* E. coli* Infection and Enumeration

After two weeks of being on a basal or supplemented diet, mice were then infected with 10^8^ CFU ETEC SEC470 [13]. At 7 day after infection, all the mice were killed by CO_2_ asphyxiation. Jejunal tissues were homogenised, serially diluted, and then plated onto MacConkey agar with the antibiotic gentamicin (40 *μ*g/mL) and tetracycline (50 *μ*g/mL). The jejunal contents and faeces were weighted and then suspended in PBS buffer. Then serial dilutions were placed on MacConkey agar treated with gentamicin (40 *μ*g/mL) and tetracycline (50 *μ*g/mL). After 24 hours, the bacterial colonies were tallied. PCR was conducted to verify the identity of the bacteria isolated. The primers used (5′-CTGTATACGTGGCAG-3′) and (5′-ACTATGGTGAATGCTCAC-3′) were obtained from ETEC* fedF* gene (GenBank accession number Z26520). The other jejunal tissues, jejunal contents, and faecal samples were collected and stored at −80°C until required.

## 4. RT-PCR and ELISA Analysis of Cytokines

After mRNA was extracted using TRIZOL reagent (Invitrogen, USA), cDNA was reverse transcribed. RT-PCR was performed according to Xiao et al. [14]. The mRNA levels of* IL-1β, IL-6, IL-17, IL-18,* and* TNF-α* were analysed with GAPDH as the reference gene. The protein levels of* IL-1β, IL-6, IL-17, IL-18,* and* TNF-α* were analysed according to Ren et al. [15] with ELISA kit from eBioscience, CA, USA.

## 5. Immunoblotting of TLR-4

To measure the level of TLR-4, an appropriate TLR-4 assay kit was used (Cayman Chemical Company, MI, USA). Equal quantities of proteins collected from jejunal tissues were separated using SDS-PAGE. Then the samples were analysed according to the method described by a previous report [16]. Using *β*-actin protein as a reference, the intensity of the signal was measured digitally.

## 6. Statistical Analyses

The data are presented as means ± standard error of the mean (SEM). All the statistical analyses were undertaken using SPSS 22.0 software (Chicago, IL, USA). The Student's* t*-test was used to analyse data differences between the control and intervention groups. Difference level at *P* < 0.05 is considered significant.

## 7. Results

**Figure 1:**
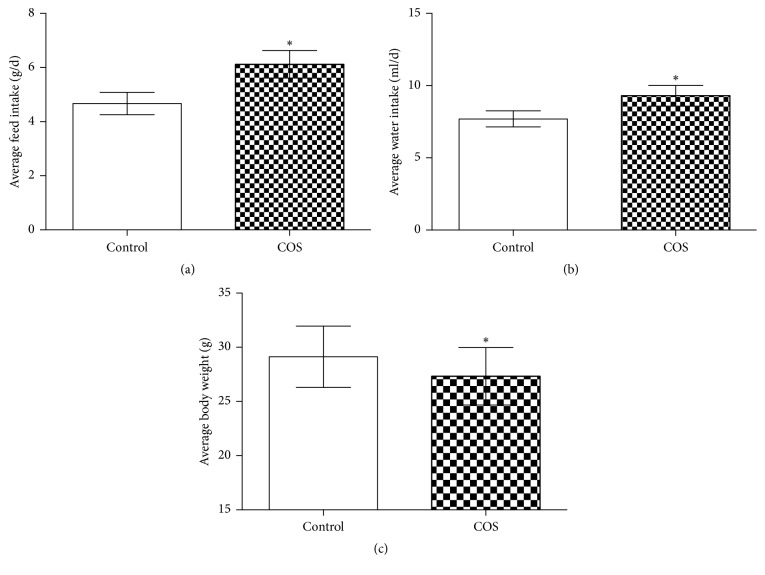
Chitosan supplementation lowers mouse body weight. (a) Average feed intake in the control group and COS group. (b) Average water intake in the control group and COS group. (c) Average body weight in the control group and COS group. *∗* indicates a significant difference between the control and COS groups (*P* < 0.05) (*n* = 10).

**Figure 2:**
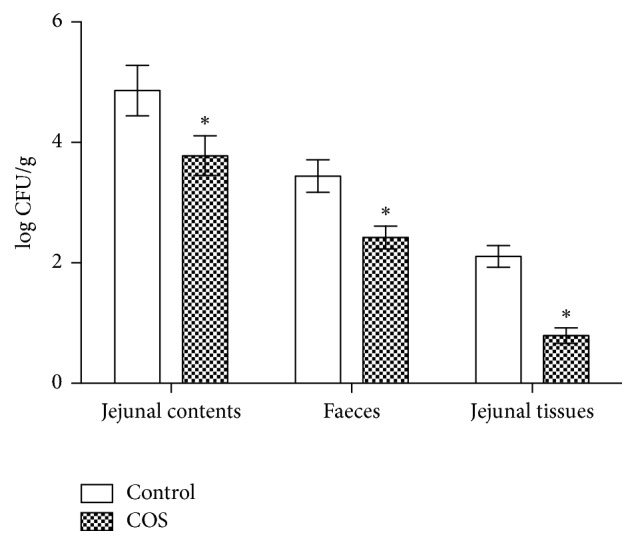
ETEC load at d 7 after infection from jejunal tissues, jejunal contents, and faeces of infected ICR mice (*n* = 10). *∗* indicates a significant difference between the control and COS groups (*P* < 0.05).

Over the two-week period, the average feed and water intake was higher in the in chitosan-supplemented mice than in control mice (*P* < 0.05) (Figures 1(a) and 1(b)). Despite this, the body weight of mice receiving the chitosan supplement was significantly lower than that of control group (*P* < 0.05) (Figure 1(c)). During the experiment, no diarrhoea was observed. At 7 days after infection, in the COS group, the ETEC loads in the jejunal contents, faeces, and jejunal tissues were significantly lower (*P* < 0.05) than that in the control group (Figure 2).

**Figure 3:**
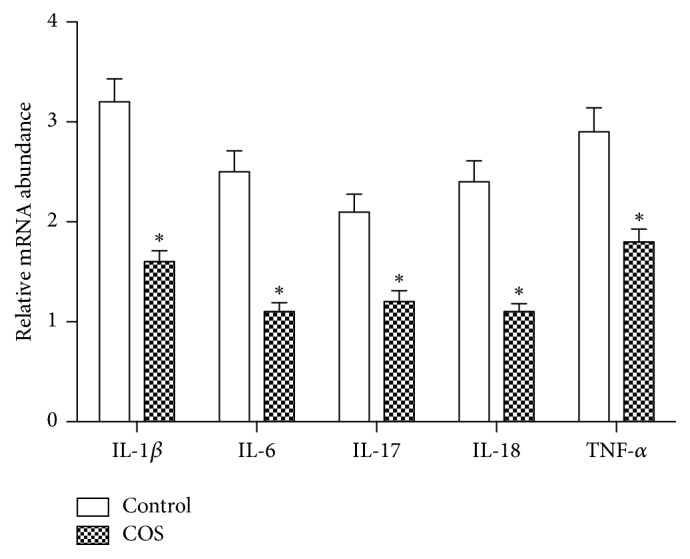
Mucosal inflammatory responses in the jejunum from the control group and COS group (*n* = 6); mRNA level of* IL-1β, IL-6, IL-17*,* IL-18*, and* TNF-α* as determined by RT-PCR. *∗* indicates a significant difference between the control and COS groups (*P* < 0.05).

Seven days after infection, the expression of* IL-1β, IL-6, IL-17*,* IL-18,* and* TNF-α* mRNA was significantly lower in the jejunum of COS-fed mice compared to those of controls (Figure 3). The ELISA results confirmed the trend with IL-1*β*, IL-6, IL-17, IL-18, and TNF-*α* being significantly lower in COS mice (Table 1).

**Figure 4:**
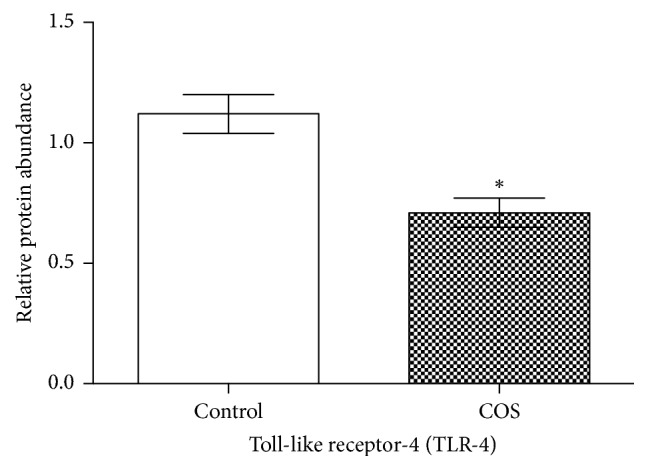
TLR4 protein level in jejunal mucosa in the control group and COS group (*n* = 6). *∗* indicates a significant difference between the control and COS groups (*P* < 0.05).

As Figure 4 indicates in the COS group the TLR4 protein level in the jejunum was significantly lower (*P* < 0.05) than that of the control group.

## 8. Discussion

Numerous studies have suggested that dietary supplements of chitosan can reduce body weight and may have applications for weight management and obesity [7, 17]. During the experimental period, the mice receiving 10^8^ CFU ETEC SEC470 did not suffer any diarrhoea. Many researches showed that the mice challenged by ETEC would not suffer from diarrhoea; however, ETEC could colonize the intestine and promote the inflammatory responses [18]. Thus we further investigated the body weight of the mice. The results demonstrate that mice fed a chitosan-supplemented diet experienced a reduction in body weight. One mechanism that has been proposed for this is that chitosan reduces the postprandial ratios of apolipoprotein B (apoB) isoforms, low-density lipoprotein cholesterol, and high-density lipoprotein. This is based on the findings of a study of the effects on lipid metabolism in glucose-tolerant rats receiving a high-sucrose diet [19]. The chitosan particles bind with cholesterol and fatty acids to form clusters in the gastrointestinal tract, thereby reducing lipid absorption [20]. In addition to its effect on lipid absorption, chitosan has been described as reducing blood glucose levels and being capable of inhibiting the carbohydrate hydrolysing enzymes maltase, sucrose, and sucrose-isomaltase in the gut [21, 22].

The findings from this study revealed that, compared to the control group, the COS mice had a significantly lower load of ETEC bacteria. According to the report of Tayel, chitosan may inhibit the growth of bacteria such as* E. coli*, Enterobacteriaceae, and* Staphylococcus aureus* [23]. As COS is a D-glucosamine oligomer, it is resistant to digestive enzyme degradation. COS may survive digestive enzymes to reach the jejunum where it interferes with the adhesion of ETEC to intestinal epithelial cells.

In this study the mRNA and protein levels of jejunal IL-1*β*, IL-6, TNF-*α*, and TLR-4 were observed in response to a chitosan-supplemented diet in mice. Intestinal epithelial cells express cytokine and chemokine receptors as well as toll-like receptors, such as TLR-4 [16]. TLR4 recruits the adaptor protein, MyD88, and TIRAP to activate NF-*κ*B, which in turn induces expression of signalling genes such as TNF-*α*, IL-1, and IL-6 [18]. Raised TLR-4 expression in intestinal epithelial cells is associated with an enhanced mucosal inflammatory response and subsequent dysfunction of the intestinal epithelial barrier [18]. Our study revealed suppressed expression of TLR-4 and consequently the mRNA and protein levels of IL-1*β*, IL-6, and TNF-*α* were correspondingly lower. The anti-inflammatory effects of COS on mice are proposed to arise from the COS inhibition of intestinal ETEC infection. It is interesting in our previous research, the mRNA levels of IL-1*β*, and IL-6 in the ETEC-challenged piglets consuming 300 mg/kg COS diet were significantly higher than those in the control group [6]. This discrepancy may be due to two reasons: firstly, it may take more times for complex network of cytokines in piglets to respond to the COS diet; thus the IL-1*β* and IL-6 mRNA levels in jejunal mucosa were still high during the sampling; secondly, the samples used for mRNA analysis are different, the jejunal mucosa was used for analysis in piglets model and the jejunal tissues were used in the mice model.

The findings here also indicated that the jejunal expression of IL-17 and IL-18 was reduced by the chitosan supplement. IL-17, manufactured in response to inducible (iTh17) and natural (nTh17) T helper cells, is a proinflammatory cytokine that recruits monocytes and neutrophils to areas of inflammation; it also stimulates local chemokine synthesis. It has been implicated in a number of autoimmune diseases [24]. On the other hand, IL-18, another proinflammatory cytokine, is strongly implicated in antitumour responses and host defence. Gene therapy studies that increase of IL-18 has been shown to provide test animals with protection against infection, tumour growth, and metastases. However, overexpression of IL-18 has been shown to result in emphysematous lesions in mice [25]. Chitosan lowered the bacterial load in jejunum, and the lower ETEC load may decrease intestinal expression of cytokines.

To summarise, chitosan supplements decrease the body weight of mice and are an effective prophylactic against in vivo infection of enterotoxigenic* E. coli*. The results presented here indicate that COS decreased the bacterial load, TLR-4, and cytokine biosynthesis. Further development of COS may provide an effective method to promote intestinal health and protection against enterotoxigenic* E. coli* infection.

## Figures and Tables

**Table 1:** Jejunal inflammatory responses in jejunum tissues from the control group and COS group (*n* = 6) and protein levels of IL-1*β*, IL-6, IL-17, IL-18, and TNF-*α* were determined by ELISA.

	Control	COS
IL-1*β* (pg/ml)	110.48 ± 8.31	67.59 ± 4.67^*∗*^
IL-6 (pg/ml)	98.45 ± 6.38	42.36 ± 3.58^*∗*^
IL-17 (pg/ml)	216.39 ± 11.23	117.96 ± 9.67^*∗*^
IL-18 (pg/ml)	182.64 ± 13.62	98.63 ± 8.45^*∗*^
TNF-*α* (pg/ml)	136.25 ± 7.12	86.23 ± 8.31^*∗*^

*∗* indicates a significant difference between the control and COS groups (*P* < 0.05).
